# 9G DNAChip Technology: Self-Assembled Monolayer (SAM) of ssDNA for Ultra-Sensitive Detection of Biomarkers

**DOI:** 10.3390/ijms14035723

**Published:** 2013-03-12

**Authors:** Satish Balasaheb Nimse, Keum-Soo Song, Junghoon Kim, Danishmalik Rafiq Sayyed, Taisun Kim

**Affiliations:** 1 Institute for Applied Chemistry and Department of Chemistry, Hallym University, Chuncheon 200-702, Korea; E-Mails: satish_nimse@hallym.ac.kr (S.B.N.); danishrs@gmail.com (D.R.S.); 2 Biometrix Technology, Inc. 202 BioVenture Plaza, Chuncheon 200-161, Korea; E-Mails: hanlimsk@empas.com (K.-S.S.); hunsjung@nate.com (J.K.)

**Keywords:** self-assembly, SAM of ssDNA, biomarker, 9G DNAChip, hybridization, C-reactive protein

## Abstract

A 9G DNAChip obtained by allowing the formation of a self-assembled monolayer (SAM) of oligonucleotides appended with nine consecutive guanines on the chip surface has been applied in the detection of biomarkers. Using a 9G DNAChip, biomarker in the concentration range of 4 pg/mL to 40 fg/mL can be easily differentiated in the buffer matrix. Moreover, it is the first time that a biomarker with a concentration of 40 fg/mL has been detected in a mixture of proteins without use of any signal amplification technique.

## 1. Introduction

The discovery of protein biomarkers whose change in expression level or state correlates with the progression of a disease is becoming increasingly important. Once validated, the proposed biomarkers can be involved in achieving an earlier diagnosis, differentiating between disease types with greater accuracy, and assessing response to treatment. Therefore, protein detection with high specificity and sensitivity is required [1]. Unfortunately, the most widely used techniques for detection of biomarkers such as mass spectrometry [2], 2-D western blotting [3], 2-D gel electrophoresis [4], enzyme-linked immunosorbent assay (ELISA) [5,6] are not suitable for the analysis of large numbers of samples or the multiplexed detection of many targets within an individual sample. The protein microarrays produced by direct immobilization methods are known to suffer from drawbacks like instability of the immobilized proteins, thus resulting in low sensitivity [7,8].

An excellent and efficient alternative is DNA microarray-based technologies [9,10]. The DNA microarrays are produced by allowing the single stranded DNA (ssDNA) molecules to form the self-assembled monolayer (SAM) on the chip surface. One of the main steps towards the development of an efficient DNA microarray for biomarker detection is to control the immobilization of the DNA probes on the surface. Factors such as surface density, orientation and conformation of the immobilized DNA probes can affect the DNA microarray performance.

The SAM of ssDNA on chip surfaces are obtained by DNA immobilization techniques such as physical adsorption [11], covalent immobilization [12,13], and streptavidin-biotin immobilization [14]. The common disadvantage of these methods is the low sensitivity and specificity due to the random arrangement of the high surface density of immobilized DNAs, leading to low hybridization yield, and non-specific interactions. The ability to routinely measure protein targets at femtomolar concentrations and lower would be extremely valuable for early disease diagnosis [15,16]. However, conventional microarray methods involving the single fluorophore labeling of either the protein target or a secondary probe molecule typically have a detection limit in the low- to mid-picomolar range [17,18].

Earlier, we reported that, these problems can be solved by immobilizing the oligonucleotides appended with nine consecutive guanines (9G probes) on the AMCA-1,3-dialdehyde (AMCA) slide to obtain a SAM ssDNA to generate a 9G DNAChip [19]. The AMCA slides are obtained by reaction of the amine modified slide glass with the AMCA to generate a monolayer of AMCA on the chip surface. The 9G probes are immobilized by multiple interactions of the nine consecutive guanines on the AMCA monolayer. The lateral spacing between the immobilized probes endowed by the nine consecutive guanines provides the high accessibility leading to more than 90% hybridization efficiency in 30 min at 25 °C [20–22].

Here, the 9G DNAChip obtained by allowing the formation of SAM of oligonucleotides appended with nine consecutive guanines on the chip surface is applied for the detection of biomarkers. Earlier we reported that the biomarkers at picogram concentrations can be measured using the DAGON method [23]. However, in this article the optimization of the biomarker detection method for the detection of the biomarkers with concentrations in the femtogram range is explained. The normal level of the CRP antigen is more than 1 ng/mL in the serum. Hence to detect antigen concentrations below 400 pg/mL to 4 fg/mL, we used buffer matrix resembling the serum.

**Scheme 1:**
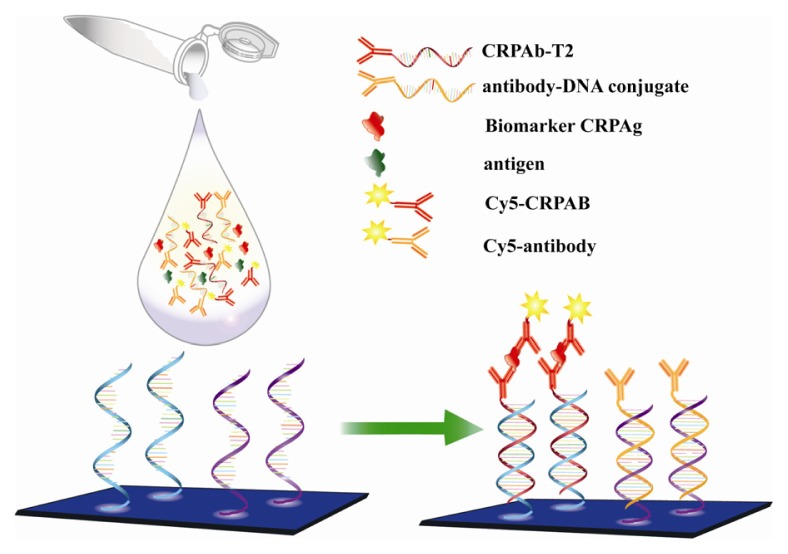
Detection of biomarkers on a 9G DNAChip. The biomolecular complex of the Cy5-labeled secondary antibody, the antibody-DNA conjugate and the target antigen (Cy5-CRPAB-CRPAg-CRPAb-T2) are allowed to form in the solution. The sequence of the target DNA T2 in the biomolecular complex is complementary to the probe immobilized on the 9G DNAChip. Hence, the biomolecular complex is site-specifically guided to the predestined area on the chip surface and hybridized at room temperature.

A significant increase in detection sensitivity without the use of any signal amplification method could be achieved if the following crucial improvements were made: (i) a substantial increase in the ratio of Cy5 labeled secondary antibodies to biomarkers, (ii) to allow the formation of biomolecular complex (the complex between the DNA labeled antibody, biomarker, and Cy5 labeled secondary antibody) in the solution, and (iii) the hybridization of complementary oligonucleotide in the biomolecular complex with the probe on the DNA chip surface as depicted in the Scheme 1, leading to more frequent biomolecular complex-probe binding.

The normal level of C-reactive protein (CRP) is more than 1 ng/mL in serum. Hence to detect biomarker concentrations below 1 pg/mL to 40 fg/mL, we used the buffer matrix resembling the serum. Scheme 1 depicts the detection of the CRP antigens by the 9G DNAChip. The biomolecular complex of the Cy5-labeled secondary antibody, the antibody-DNA conjugate and the target antigen formed in the solution are site-specifically guided to the predestined area on the chip surface and hybridized at room temperature.

## 2. Results and Discussion

Recently, we reported 9G DNAChips based on 9G DNAChip technology, which show more than 80% hybridization efficiency at 25 °C in 5min with specificity more than 97%. The 9G DNAChips used here were produced by immobilization of the Probe1–Probe11 (see the Supplementary Information Table S1, Scheme S1) following the reported method [24]. The CRP antibody (CRPAb) was conjugated with the oligonucleotide target probe T2 (see the Supplementary Information Table S1). The secondary CRP antibody (CRPAB) was labeled with Cy5 dye. The biomarker CRP antigen (CRPAg) was used to develop the method for ultra-sensitive detection of biomarkers (see the Supplementary Information for abbreviations).

It is well known that, biomolecular complexes (Cy5-CRPAB-CRPAg-CRPAb-T2) between the biomarker and its specific antibodies are formed spontaneously upon mixing in the solution phase. However, the concentration of both the DNA labeled capture antibody (CRPAb-T2) and the Cy5 labeled secondary antibody (Cy5-CRPAB) plays a vital role in the formation of the biomolecular complex (Cy5-CRPAB-CRPAg-CRPAb-T2) in the presence of the biomarker (CRPAg).

Therefore, to find the optimum concentration of the DNA labeled capture antibody (CRPAb-T2), the hybridization mixtures were prepared by mixing the Cy5-labeled CRP secondary antibody (Cy5-CRPAB) with a concentration of 1 μg/mL and CRP antibody-DNA conjugate (CRPAb-T2) with concentrations ranging from 8 μg/mL to 65 ng/mL in the hybridization solution. On addition of the CRPAg (4 ng/mL–400 fg/mL) to the mixture of the Cy5-CRPAB, CRPAb-T2, a Cy5-CRPAB-CRPAg-CRPAb-T2 complex is formed in the solution. The target oligonucleotide Cy5-T1 complementary to the immobilized Probe11 (hybridization control (HC) probe) was also added to the hybridization solution.

**Figure 1:**
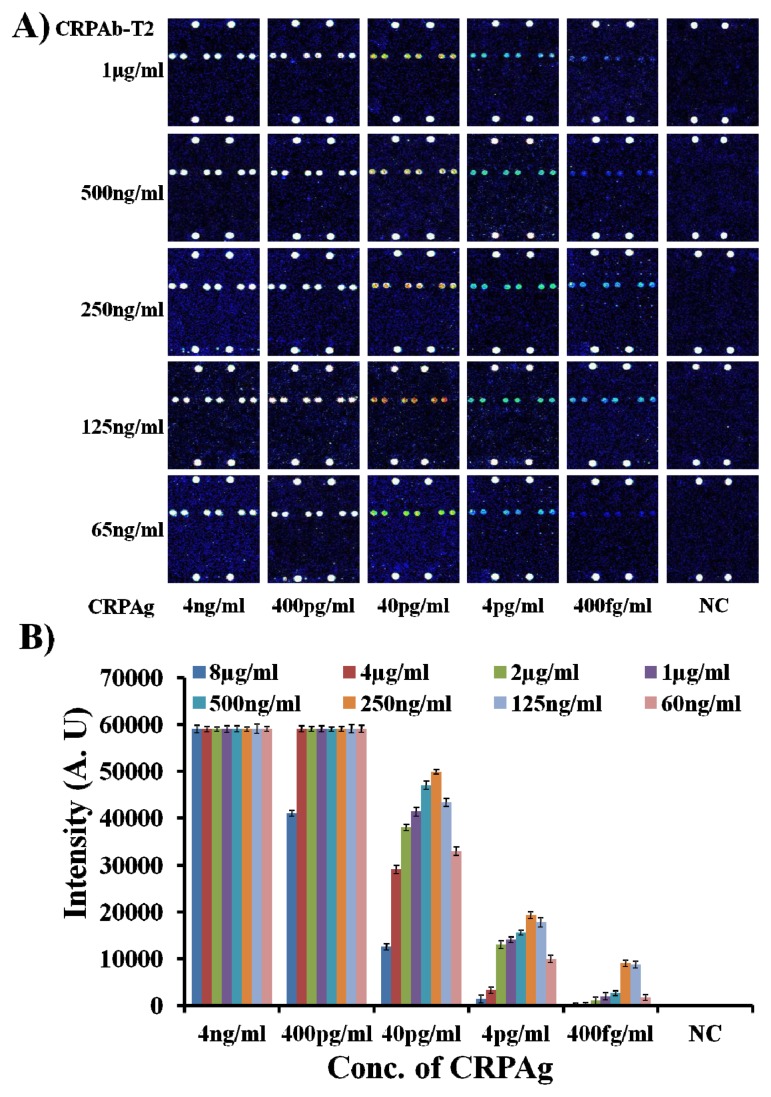
Optimum concentration of DNA labeled capture antibody (CRPAb-T2) (**A**) Fluorescence images for the detection of the biomarker CRP antigen (CRPAg) (4 ng/mL–400 fg/mL) by using CRPAb-T2 with concentrations of 1 μg/mL–65 ng/mL; (**B**) Corresponding graph representing the fluorescence intensities for the detection of CRPAg, the NC indicates negative control, PMT gain = 90.

The 50 μL of the hybridization mixture was loaded in the hybridization chambers formed by covering the 9G DNAChips with Secure-Seal™ and then allowed to hybridize for 60 min at 25 °C. After loading the hybridization mixture on the 9G DNAChip surface, the target DNA T2 in the biomolecular complex, guide the biomolecular complex to the specific site and hybridize with the surface-bound complementary oligonucleotide Probe2 for the detection (for the detailed preparation of the hybridization mixture please see the Supplementary Information Table S2). After washing and drying, the 9G DNAChips were scanned and analyzed. The obtained results are presented in Figure 1.

As shown in Figure 1A, the biomolecular complex (Cy5-CRPAB-CRPAg-CRPAb-T2) of CRPAg with Cy5-CRPAB and CRPAb-T2 is site-specifically guided and hybridized to the complementary Probe2 on the chip surface. In a similar way, the target probe Cy5-T1 is hybridized to the complementary Probe11 on the chip surface, indicating the hybridization control. Figure 1 clearly demonstrates that the DAGON method allows the detection of antigens with a concentration as low as 400 fg/mL. However, Figure 1 also depicts the effect of the concentration of the DNA labeled capture antibody (CRPAb-T2) (see the Supplementary Information Figure S1) on the detection of the biomarker CRPAg. Figure 1 clearly demonstrates that the decrease in the concentration of the capture antibody CRPAb-T2 increases the detection limit. The fluorescence intensities of the biomolecular complexes formed by the use of 8 μg/mL–1 μg/mL and 65 ng/mL of CRPAb-T2 and 400 fg/mL of CRPAg and hybridized with the immobilized probe on the chip surface, are 80–90 times lower than those with the use of 125 ng/mL–250 ng/mL of CRPAb-T2 and 400 fg/mL of CRPAg (Figure 1, see the Supplementary Information Figure S1). The hybridization pattern of the biomolecular complexes formed by using different concentrations of the capture antibody CRPAb-T2 suggests that 250 ng/mL is the optimum concentration for the efficient detection of 400 fg/mL of the biomarker CRPAg. One of the possible reasons behind this hybridization pattern is that use of hybridization mixture with the higher concentration of the CRPAb-T2 leads to a much higher amount of the free CRPAb-T2 than the CRPAb-T2 in the biomolecular complex Cy5-CRPAB-CRPAg-CRPAb-T2. Therefore the free CRPAb-T2 competes with the CRPAb-T2 in the biomolecular complex Cy5-CRPAB-CRPAg-CRPAb-T2 to hybridize with the immobilized Probe2, which leads to the lower fluorescence intensities and significantly hampers the limit of detection of the biomarker CRPAg.

**Figure 2:**
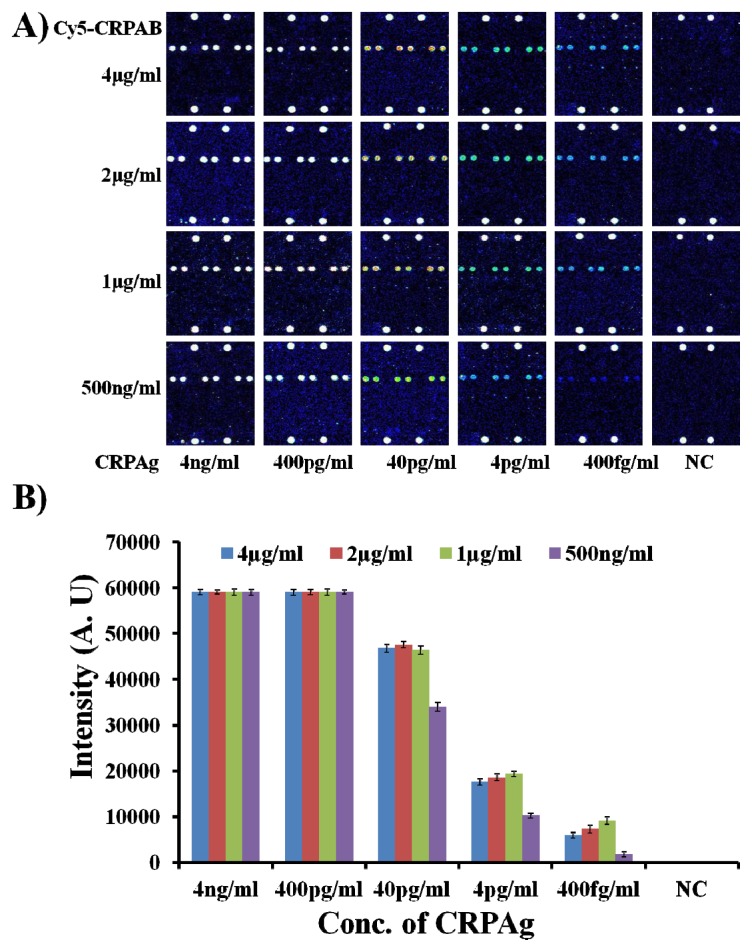
Optimum concentration of Cy5 labeled secondary antibody (Cy5-CRPAB) (**A**) Fluorescence images for the detection of CRPAg (4 ng/mL–400 fg/mL) by using Cy5-CRPAB at concentrations of 4 μg/mL–500 ng/mL; (**B**) Corresponding graph representing the fluorescence intensities for the detection of CRPAg, the NC indicates negative control, PMT gain = 90.

With the optimum concentration of the capture antibody CRPAb-T2 being defined as 250 ng/mL, it was necessary to find the optimum concentration of the Cy5 labeled secondary antibody (Cy5-CRPAB) as it also plays a vital role in the formation of the biomolecular complex (Cy5-CRPAB-CRPAg-CRPAb-T2) in the presence of the biomarker (CRPAg). Therefore, to find the optimum concentration of the Cy5 labeled secondary antibody (Cy5-CRPAB), the hybridization mixtures were prepared by mixing CRPAb-T2 with a concentration of 250 ng/mL and Cy5-CRPAB with concentrations ranging from 4 μg/mL to 500 ng/mL in the hybridization solution. On addition of CRPAg (4 ng/mL–400 fg/mL) to the mixture of the Cy5-CRPAB, CRPAb-T2, they together form a Cy5-CRPAB-CRPAg-CRPAb-T2 complex in the solution. The 50 μL of the hybridization mixture was loaded in the hybridization chambers formed by covering the 9G DNAChips with Secure-Seal™, and then allowed to hybridize for 60 min at 25 °C (for the detailed preparation of the hybridization mixture please see the Supplementary Information Table S3). After washing and drying, the 9G DNAChips were scanned and analyzed. The obtained results are presented in Figure 2.

Figure 2 clearly depicts the effect of the concentration of the Cy5 labeled secondary antibody (Cy5-CRPAB) on the detection of the biomarker CRPAg. Figure 2 also clearly demonstrates that the decrease in the concentration of the Cy5-CRPAB from 4 μg/mL to 1 μg/mL decreases the fluorescence intensity for the detection of the CRPAg from 4 ng/mL to 400 fg/mL. The fluorescence intensities of the biomolecular complexes formed by the use of 4 μg/mL–1 μg/mL of Cy5-CRPAB and 400 fg/mL of CRPAg upon hybridization with the immobilized probe on the chip surface are almost similar. However, these fluorescence intensities are 4–5 times higher than the biomolecular complexes formed by the use 500 ng/mL of Cy5-CRPAB and 400 fg/mL of CRPAg upon hybridization with the immobilized probe on the chip surface. The hybridization pattern of the biomolecular complexes formed by using different concentration of the Cy5 labeled secondary antibody (Cy5-CRPAB) suggests that a concentration of 4 μg/mL–1 μg/mL gives efficient detection for 400 fg/mL of the biomarker CRPAg. However, as the use of 4 μg/mL–1 μg/mL of Cy5-CRPAB showed similar results we considered 1 μg/mL of the Cy5-CRPAB as an optimum concentration for the detection of the biomarker CRPAg.

The 1 μg/mL of the Cy5-CRPAB and 250 ng/mL of the CRPAb-T2 were the optimum concentrations for the efficient detection of 400 fg/mL of the biomarker CRPAg upon hybridization for 60 min on the 9G DNAChip surface. Further, it was important to investigate the effect of the hybridization time on the detection of the biomarker CRPAg using the proposed method.

**Figure 3:**
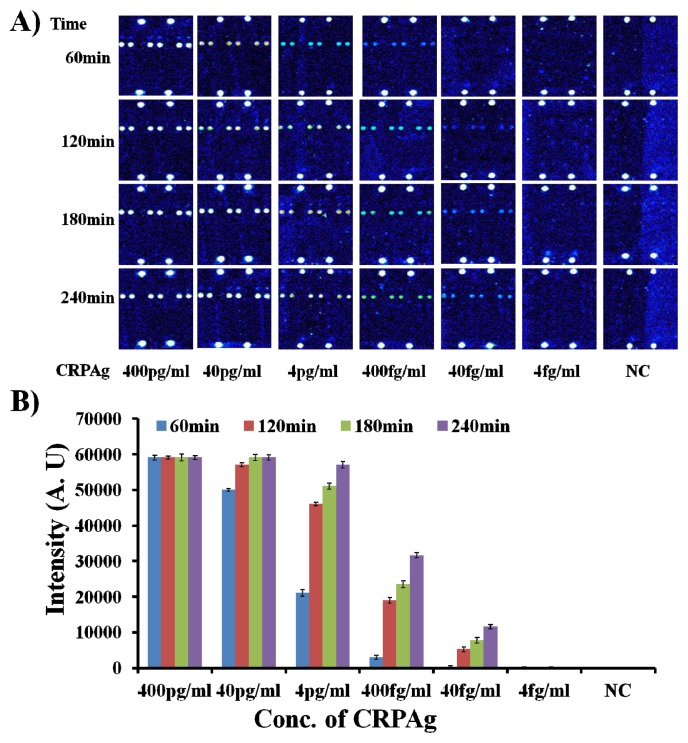
Optimum time for the hybridization of biomolecular complex (Cy5-CRPAB-CRPAg-CRPAb-T2) with the probe on the 9G DNAChip (Cy5-CRPAB) (**A**) Fluorescence images for the detection of CRPAg (400 pg/mL–4 fg/mL) using Cy5-CRPAB (1 μg/mL) and CRPAb-T2 (250 ng/mL); (**B**) Corresponding graph representing the fluorescence intensities for the detection of CRPAg, the NC indicates negative control, PMT gain = 90.

Therefore, the hybridization mixtures were prepared by mixing CRPAb-T2 at a concentration of 250 ng/mL, Cy5-CRPAB at a concentration of 1 μg/mL, and CRPAg with concentration ranging from 400 pg/mL to 4 fg/mL) (for the detailed preparation of the hybridization mixture please see the Supplementary Information Table S4). The 50 μL of the hybridization mixture was loaded in the hybridization chambers formed by covering the 9G DNAChips with Secure-Seal™, and then allowed to hybridize for different time intervals of 60 min, 120 min, 180 min, and 240 min at 25 °C, respectively. After washing and drying, the 9G DNAChips were scanned and analyzed. The obtained results are presented in Figure 3.

As shown in Figure 3, the biomarker CRPAg with concentration of 400 fg/mL can be efficiently detected after hybridization for 60 min. Interestingly, upon increase in the hybridization time from 60 min to 120–240 min, there is a sharp increase of 7–11 times in the fluorescence intensity. However, fluorescence intensity with 240 min hybridization is only twice that of the fluorescence intensity with 120 min hybridization.

Figure 3 also demonstrates that the biomarker CRPAg with concentration of 40 fg/mL can be efficiently detected after hybridization for 120 min–240 min, but the fluorescence intensity with 240 min hybridization is more than two times higher than the fluorescence intensity with 120 min hybridization. This indicates that the longer hybridization time helps in the detection of the biomarker at the sub-femtogram level.

However, the detection limits of the protein microarrays are significantly limited due to the signals for non-specific interactions [25]. If false signals for non-specific interactions are minimized, any signals higher than that of NC can be used for effective detection of antigens. Therefore, it is very important to be sure that there are no non-specific interactions in the detection of the biomarkers at the sub-femtogram level.

The Figures 1B, 2C, and 3 demonstrate the fluorescence maps for the detection of the CRPAg at 4 ng/mL–40 fg/mL and NC (without CRPAg). The fluorescence intensities of the spotted area of NC and the background (non-spotted area) in each case are around 6200–6500. Hence, the similar fluorescence intensities for the spotted areas and the background indicate that there are no non-specific interactions. It is also important to notice that the fluorescence intensity for the CRPAg at 40 fg/mL after hybridization for 120 min–240 min is in the range of 5,000–11,550. Therefore, the presented method shows the efficient detection of the 40 fg/mL of biomarker CRPAg with 100% specificity. There were no measurable hybridization signals from the spots of the non-complementary probes (Probe1, Probe3-11), indicating that nonspecific hybridization did not occur. The major reasons for such a high sensitivity and specificity could be the formation of the biomolecular complex in the solution, and the hybridization, washing, and drying at room temperature.

Typically ELISA [26] has been widely used for the detection and quantification of CRP biomarkers. Although ELISA is a long standing standard for quantitative analysis of CRP, it suffers from a relatively low throughput because of its lack of multiplexing ability and high reagent and sample consumption [27]. The accuracies of traditional ELISAs vary considerably when the sample concentrations are lower than 1 ng/mL. High-sensitivity ELISAs (hs-ELISAs) improve the detection sensitivity to as low as 0.014 μg/mL by signal amplification [28]. Protein microarrays obtained by direct immobilization of capture antibodies were developed in order to overcome the limitations of the conventional ELISA based method. However, these microarrays must have relatively large sample volumes and additional capture materials. Additionally, these microarrays have a high limit of detection (LOD) and a long incubation time [29]. DNA-Directed Immobilization (DDI) was employed to improve the stability of proteins by immobilizing them on the surface shortly before the detection of antigens [30]. In such stepwise methods, first the proteins are immobilized on the surface and in the second step they are allowed to react with the target proteins. The disadvantage of this method is that once the proteins are immobilized on the surface they still have a chance to lose their activity over a period of time [31]. Thus, methods like DDI improve the stability of proteins, but the lengthy process limits the sensitivity of the method to 100 pg/mL [15].

According to the proposed method, the CRP antigens in the concentration range of 40 fg/mL to 4 pg/mL can be easily differentiated in the buffer matrix. Moreover, it is the first time that antigen with concentrations of 40 fg/mL has been detected without use of any amplification technique.

## 3. Experimental Section

### 3.1. Labeling of Oligonucleotide, Antibody and the Preparation of Protein-DNA Conjugate

#### 3.1.1. Labeling of Oligonucleotides and Proteins with Cy5 Dye

Briefly, the Cy5-T1, and the Cy5 labeled CRP monoclonal secondary antibody (Cy5-CRPAB) were obtained by the reaction of the amine functions in the amine modified oligonucleotides (T1, T2) and the amine function in the CRPAB antibody with the Cy5Dye mono-reactive NHS ester, respectively, following the standard protocol provided by the manufacture with the mono-reactive Cy5Dye™ (GE Healthcare UK Limited, Buckinghamshire, UK).

#### 3.1.2. Synthesis and Purification of Protein-DNA Conjugates

The CRPAb-T2 was synthesized by the reaction of CRPAb with the slfo-SMCC activated oligonucleotide target probe T2, following the reported method [32].

#### 3.1.3. Preparation of a Biomolecular Complex (Cy5-CRPAB-CRPAg-CRPAb-T2)

The biomolecular complex Cy5-CRPAB-CRPAg-CRPAb-T2 was obtained by mixing 5 μL of CRPAb-T2 conjugate (0.65 μg/mL–80 μg/mL), 5 μL of Cy5-CRPAB (10 μg/mL), 20 μL of CRPAg (1 pg/mL–10 ng/mL), and 5 μL of Cy5-T1 (40 fmol/mL) in 15 μL of hybridization solution for 5 min and used immediately.

### 3.2. General Hybridization Procedure

Each hybridization chamber of the 9G DNAChip was covered with 50 μL of the hybridization mixture and then incubated at 25 °C for 60 min or more unless otherwise stated. Then the 9G DNAChip was rinsed with washing buffer solutions A and B successively for 2 min each in order to remove the excess target DNA and dried with a commercial centrifuge (1000 rpm). The fluorescence signal of the microarray was measured on a ScanArrayLite, and the images were analyzed by Quant Array software.

## 4. Conclusions

In this article, we have presented the application of a 9G DNAChip for detection of biomarkers at sub-femtogram concentrations. The 9G DNAChip was obtained by allowing the formation of self-assembled monolayer (SAM) of single stranded DNA (ssDNA) on the chip surface. According to the proposed method, biomarker with a range of concentrations of 4 pg/mL to 40 fg/mL can be easily differentiated. Moreover, it is the first time that biomarker with a concentration as low as 40 fg/mL has been detected in a mixture of proteins without the use of any signal amplification technique. This represents an improvement of 1000 times in sensitivity, compared to previously reported methods. The proposed method can be applied for the efficient detection of multiple biomarkers for diagnostic applications. The ability to perform rapid, multiplexed protein biomarker analyses in complex biological fluids will undoubtedly lead to new opportunities for the understanding and treatment of disease. Hence, the presented method is under investigation for the detection of biomarkers in clinical samples.

## Supplementary Information
