# Supplementary material for: 9G DNAChip Technology: Self-Assembled Monolayer (SAM) of ssDNA for Ultra-Sensitive Detection of Biomarkers
Source: Int J Mol Sci. 2013 Mar 12;14(3):5723–33. doi: 10.3390/ijms14035723 (PMC3634481; doi:10.3390/ijms14035723)
Supplement: Supplementary file 1 [file ijms-14-05723-s001.pdf]

Supplementary Information

1. Materials

All chemicals were purchased from Sigma-Aldrich Chemicals, Korea. All the oligonucleotides were purchased from Bioneer, Korea. For labeling of the oligonucleotides and antibodies with Cy5, the Cy5Dye mono-reactive NHS ester (PA25001) was purchased from GE Healthcare UK Limited, Buckinghamshire, UK. C-reactive protein Antigen (CRPAg) (Catalog #. 30-AC05), CRP monoclonal antibody (CRPAb) (Catalog #. 10-C33C), CRP monoclonal secondary antibody (CRPAB) (Catalog #. 10-C33B), were purchased from the Fitzgerald Industries International, USA. Glass slides (2.5 × 7.5 cm) were purchased from Paul Marienfeld GmbH & Co. KG, Germany. All washing solvents for the substrates are of HPLC grade from SK Chemicals, Korea. Ultrapure water (18 MΩ/cm) was obtained from a Milli-Q purification system (Millipore, Billerica, MA, USA). The 9G DNAChips were obtained from the Biometrix Technology Inc., Chuncheon, South Korea.

2. Instruments

Oligonucleotides were spotted using Qarray2 microarrayer (Genetix Technologies, Inc., now part of Molecular Devices, Sunnyvale, CA, USA) Hybridization was done at 25 °C using the commercial incubator and then the slides were dried using the commercial centrifuge (1000 rpm). The fluorescence signal of the microarray was measured on ScanArrayLite (Packard Bioscience now PerkinElmer, Livermore, CA, USA), and the images were analyzed by Quant Array software (Packard Bioscience now PerkinElmer, Livermore, CA, USA).

3. Abbreviations

CRP, C-reactive protein; CRPAg, C-reactive protein antigen; CRPAb, CRP monoclonal antibody; T2, Target oligonucleotide complementary to the oligonucleotide Probe2 immobilized on the 9G DNAChip; CRPAb-T2, antibody-DNA conjugate of CRPAb and target oligonucleotide T2; Cy5-CRPAB, Cy-5 dye labeled CRP monoclonal secondary antibody; Cy5-CRPAB-CRPAg-CRPAb-T2, biomolecular complex of Cy5-CRPAB, CRPAg, and CRPAb-T2; Cy5-T1, Target oligonucleotide complementary to the oligonucleotide Probe11 (hybridization control probe) immobilized on the 9G DNAChip.

4. Probes and Target Oligonucleotides

Table S1. Sequences and the nomenclature of probes and target oligonucleotides.

Capture Probes	Sequence
Probe1	5'-GGGGGGGGG CTT TAT TTT CC TAC GAC TTG GGG AGG-3'
Probe2	5'-GGGGGGGGG CTT TAT TTT CC ATC GTG TAG CGG TGG-3'
Probe3	5'-GGGGGGGGG CTT TAT TTT CG TAG GAC ATG GCC ACC-3'
Probe4	5'-GGGGGGGGG CTT TAT TTT GC TAC CTG TTG CCG TGG-3'
Probe5	5'-GGGGGGGGG CTT TAT TTT CC ACT GTT CTC GGC ACG-3'
Probe6	5'-GGGGGGGGG CTT TAT TTT CC CAT CAC TGG TGG AGG-3'
Probe7	5'-GGGGGGGGG CTT TAT TTT CT TGC GAC AAG CCC AGG-3'
Probe8	5'-GGGGGGGGG CTT TAT TTT CA CAC GCT ATC GGG TGG-3'
Probe9	5'-GGGGGGGGG CTT TAT TTT TA CAC GAC CTG CGG AGC-3'
Probe10	5'-GGGGGGGGG CTT TAT TTT CC CAT ACC TTG GGA GGG-3'
Probe11	5'-GGGGGGGGG TTT CCT AGT GGC TCT ATG GTA AC-3'
**Target DNA**	**Sequence**
Cy5-T1 (Complementary to Probe11)	3'- GGA TCA CCG AGA TAC CAT TG GAG ACT GCG -Cy5-5'
T2 (Complementary to Probe2)	3'- GG TAG CAC ATC GCC ACC TTT TTT TTT TTT-NH_2-5'
Cy5-CRPAB-CRPAg-CRPAb-T2	3'- GG TAG CAC ATC GCC ACC TTT TTT TTT TTT- CRPAb-CRPAg-CRPAB-Cy5-5'

The GGG GGG GGG sequence in the oligonucleotide probes (Probe1-Probe11) is used for the immobilization of the oligonucleotide on the 9G DNAChip. Whereas the CTT TAT TTT in the oligonucleotide probe is used as a vertical spacer. The GAG ACT GCG and TTT TTT TTT TTT sequence in the target oligonucleotides T1 and T2 respectively, are used as vertical spacers. The complementary sequences of the probes Probe11, Probe2 with the target probes T1, T2, respectively, are underlined.

5. Scheme for Immobilization of the Probes on the 9G DNAChip

Scheme S1. Scheme for immobilization of the Probes on the 9G DNAChip.

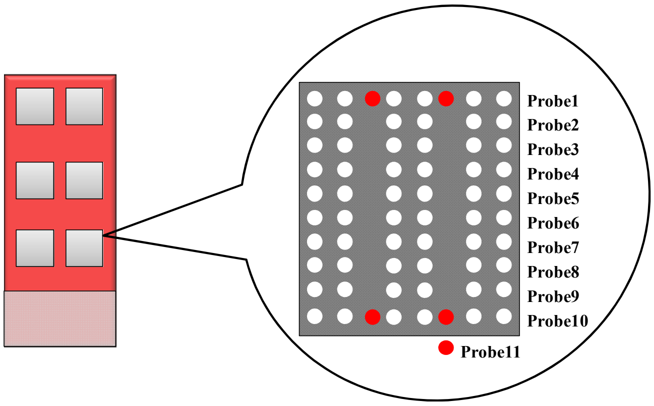

6. Composition of Used Solutions

1. Hybridization buffer (pH = 7.4): 12× SSC, 0.1% BSA, 5 mM EDTA, 0.05% NaN_3
2. Washing buffer solution A (pH = 7.4): 0.1% SDS in 4× SSC
3. Washing buffer solution B (pH = 7.4): 4× SSC

7. Hybridization Mixture

Five microliter each of the CRPAb-T2, Cy5-CRPAB, CRPAg, and Cy5-T1 were mixed in the hybridization solution to make 50 µL of final hybridization mixture.

Table S2. The final composition of the hybridization mixture to find the optimum concentration of capture antibody-DNA conjugates CRPAb-T2.

Constituents	CRPAg	NC
CRPAb-T2 (0.65 µg/mL–80 ug/mL)	5 µL	5 µL
Cy5-CRPAB (10 µg/mL)	5 µL	5 µL
CRPAg (1 pg/mL–10 ng/mL)	20 µL	-
Cy5-T1 (40 fmol/mL)	5 µL	5 µL
Hybridization solution (12× SSC, 0.1% BSA, 5 mM EDTA, 0.05% NaN_3)	15 µL	20 µL
Total Volume of the hybridization mixture	50 µL	50 µL

Table S3. The final composition of the hybridization mixture to find the optimum concentration of Cy5 labeled secondary antibody Cy5-CRPAB.

Constituents	CRPAg	NC
CRPAb-T2 (2.5 µg/mL)	5 µL	5 µL
Cy5-CRPAB (5 ng/mL–40 µg/mL)	5 µL	5 µL
CRPAg (1 pg/mL–10 ng/mL)	20 µL	-
Cy5-T4 (40 fmol/mL)	5 µL	5 µL
Hybridization solution (12× SSC, 0.1% BSA, 5 mM EDTA, 0.05% NaN_3)	15 µL	20 µL
CRPAb-T2 (2.5 µg/mL)	5 µL	5 µL
Total Volume of the hybridization mixture	50 µL	50 µL

Table S4. The final composition of the hybridization mixture to investigate the hybridization time required for the effective detection of the biomarker CRPAg.

Constituents	CRPAg	NC
CRPAb-T2 (2.5 µg/mL)	5 µL	5 µL
Cy5-CRPAB (10 ng/mL)	5 µL	5 µL
CRPAg (10 fg/mL–1 ng/mL)	20 µL	-
Cy5-T4 (40 fmol/mL)	5 µL	5 µL
Hybridization solution (12× SSC, 0.1% BSA, 5 mM EDTA, 0.05% NaN_3)	15 µL	20 µL
CRPAb-T2 (2.5 µg/mL)	5 µL	5 µL
Total Volume of the hybridization mixture	50 µL	50 µL

8. Time Dependent Hybridization Efficiency of Probe5 with Cy5-CRPAB-CRPAg-CRPAb-T2

Figure S1. Optimum concentration of DNA labeled capture antibody (CRPAb-T2). (**A**) Fluorescence images for the detection of CRPAg (4 ng/mL–400 fg/mL) by using CRPAb-T2 with the concentrations of 8 µg/mL–65 ng/mL; (**B**) Corresponding graph representing the fluorescence intensities for the detection of CRPAg, the NC indicates negative control, PMT gain = 90.

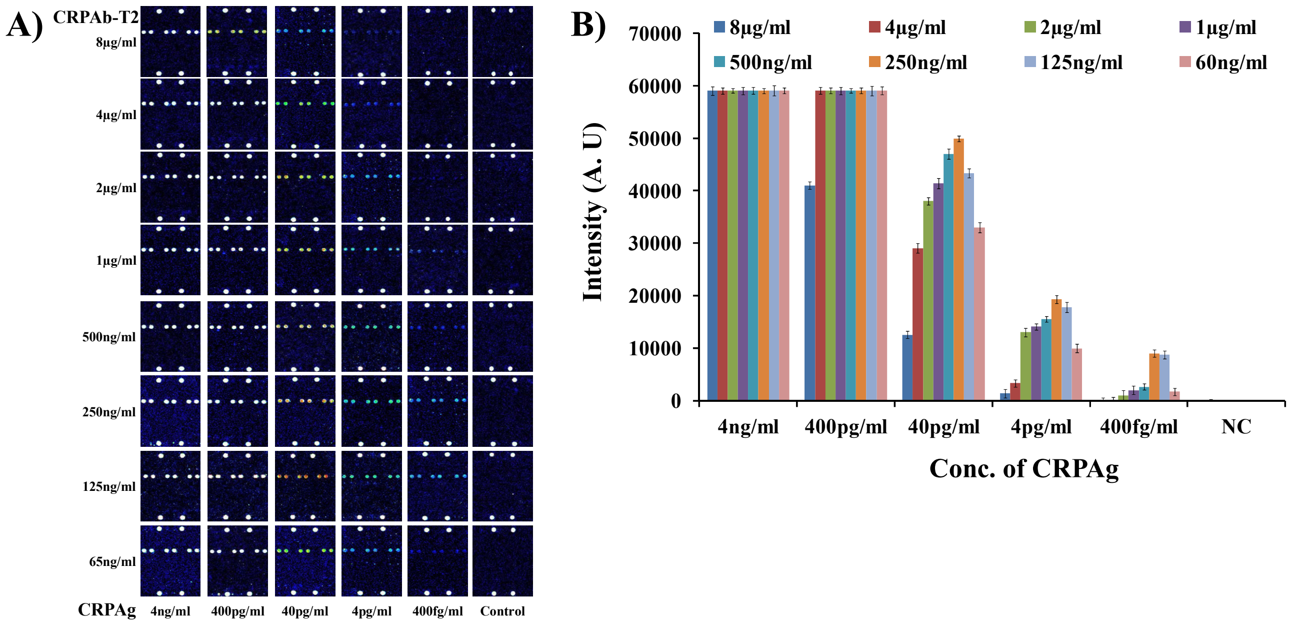

© 2013 by the authors; licensee MDPI, Basel, Switzerland. This article is an open access article distributed under the terms and conditions of the Creative Commons Attribution license (http://creativecommons.org/licenses/by/3.0/).
